# Nest-Like MnO_2_ Nanowire/Hierarchical Porous Carbon Composite for High-Performance Supercapacitor from Oily Sludge

**DOI:** 10.3390/nano11102715

**Published:** 2021-10-14

**Authors:** Xiaoyu Li, Dong Han, Zhiqiang Gong, Zhenbo Wang

**Affiliations:** 1 College of Mechanical and Electronic Engineering, Shandong University of Science and Technology, Qingdao 266590, China; 2 College of New Energy, China University of Petroleum (East China), Qingdao 266580, China; S16030382@s.upc.edu.cn (D.H.); wangzhb@upc.edu.cn (Z.W.); 3 State Grid Shandong Electric Power Research Institute, Jinan 250003, China; gongzhiqiang@upc.edu.cn

**Keywords:** supercapacitors, MnO_2_ nanowires, hierarchical porous carbon, oily sludge

## Abstract

In the aim to go beyond the performance tradeoffs of classic electric double-layer capacitance and pseudo-capacitance, composites made out of carbon and pseudo-capacitive materials have been a hot-spot strategy. In this paper, a nest-like MnO_2_ nanowire/hierarchical porous carbon (HPC) composite (MPC) was successfully fabricated by a controllable in situ chemical co-precipitation method from oily sludge waste. Due to the advantages of high surface area and fast charge transfer for HPC as well as the large pseudo-capacitance for MnO_2_ nanowires, the as-prepared MPC has good capacitance performance with a specific capacitance of 437.9 F g^−1^ at 0.5 A g^−1^, favorable rate capability of 79.2% retention at 20 A g^−1^, and long-term cycle stability of 78.5% retention after 5000 cycles at 5 A g^−1^. Meanwhile, an asymmetric supercapacitor (ASC) was assembled using MPC as the cathode while HPC was the anode, which exhibits a superior energy density of 58.67 W h kg^−1^ at the corresponding power density of 498.8 W kg^−1^. These extraordinary electrochemical properties highlight the prospect of our waste-derived composites electrode material to replace conventional electrode materials for a high-performance supercapacitor.

## 1. Introduction

Electrical energy storage systems have drawn great attention in recent decades such as conventional capacitors and batteries, Li-ion batteries, and supercapacitors [1]. Among them, supercapacitors, also referred to as electrochemical capacitors, can be seen as the primary choice for applications in energy storage systems because of their higher energy density than conventional capacitors and better power density than batteries [2]. Generally, supercapacitors can be classified into electric double-layer capacitors (EDLC) and pseudo capacitors, according to the different energy storage mechanisms. The capacitance in EDLCs is controlled by the surface electrical double layers between the electrode and electrolyte, while in pseudo capacitors, the energy is stored by the fast and reversible faradaic redox reactions in the electrode, which are controlled by diffusion. [3]. As is known to all, pseudo capacitors usually provide higher specific capacitances (C_s_) and energy densities, but suffer from some shortcomings such as poor electrical conductivity and cycling stability, while EDLCs are the opposite. The plans to realize the synergistic effect of the advantages of both EDLC and pseudo capacitor are highly desirable to improve the electrochemical performance [4].

The electrode material plays a key role in the development of high-performance supercapacitors [5]. In recently reported literatures, various candidates have been investigated including carbon materials, conducting polymers, transition metal oxides, and hydroxides [6]. Among them, transition metal oxides have attracted considerable interest because of the variety of oxidation states for efficient redox charge transfer, which can provide pseudo-capacitance effects such as Mn_x_O_y_ [7,8], Co_x_O_y_ [9], Fe_x_O_y_ [10], and Ni_x_O_y_ [11]. MnO_2_ is one of the most promising metal oxides due to its low cost, high theoretical capacitance, and environmental safety properties [12]. The capacitance can be significantly enhanced through fast faradaic redox reactions occurring on the surface of MnO_2_ [13]. However, as with other pseudo-capacitor electrode materials, MnO_2_ suffers from low surface area in bulk and poor electrical conductivity, which limits its practical capacitance application. Hence, an emerging new concept is to integrate MnO_2_ nanostructures with the conductive substrates to form a composite. Moreover, the interface interactions between the MnO_2_ nanostructure and substrates were of great influence on the capacitance performance of the composites [14].

With excellent electrical conductivity and high specific surface area, carbon nanomaterials such as graphene [15], carbon nanofibers [16], carbon nanotubes [17], and their hybrids [18,19,20] have been selected as substrate components for preparing MnO_2_-carbon composites, which not only provide stable scaffolds for MnO_2_, but also facilitate charge transport for pseudo capacitive reactions of MnO_2_ during the charge/discharge process [21,22]. However, these nano-sized composites are still limited in practical application by problems associated with their high cost. Meanwhile, given the strong van der Waals interactions, the nano-sized materials are easy to stack together during the electrode preparation [23], which may lead to sacrificing the effective surface area and the reduction in specific capacitance [24]. Recent studies have shown that 3D hierarchical nanostructured carbons are promising candidates as substrates of nanoscaled MnO_2_ accumulation to fabricate hybrid composites [25,26] because of their unique internal porous structures. For example, 3D graphene foam [27], carbon nanotube sponges [28], hierarchical porous carbon (HPC) [29,30], and hierarchical hybrid nanostructures [31,32] have been developed. Despite these achievements, it is also desirable to develop simple and scalable methods to synthesize MnO_2_/HPC composites. HPCs are usually prepared from polymer precursors using template-assisted methods. In recent years, a lot of waste materials and biomass resources such as plant fibers [30,33], animal tissues [34], or industry waste [35] have been transformed into HPCs by their self-structuring procedure. Obviously, the high value-added utilization of worthless and renewable material is beneficial to both environmental protection and sustainable development [36,37]. As the most refractory contaminants in the petroleum industry, oily sludge (OS) has become a great threat to the environment [38]. To our knowledge, few works have been carried out so far concerning about OS-based HPCs and MnO_2_ composites.

In this work, an effective approach was implemented to synthesize the MnO_2_/HPC composite material (MPC) by using OS waste. First, the HPC substrate was prepared by carbonization and the chemical activation method. Then, the obtained HPC was surface modified by an acid pre-oxidation process. Furthermore, MnO_2_ nanowires were grown on the HPC substrate using a controllable in situ chemical co-precipitation method. The MnO_2_/HPC nanostructured electrode has been used as an anode in supercapacitors and has demonstrated high capacitance and excellent cycling stability. HPC served as the mechanical support to avoid structure collapse and provided highways for electronic and ionic transport to MnO_2_ nanowires. MnO_2_ nanowires enhanced the specific capacitance by introducing reversible redox reactions at the same time.

## 2. Materials and Methods

### 2.1. Materials

The oily sludge sample used in this study was a conventional oil tank bottom sludge in the petrochemical industry. It was obtained during the tank cleaning process in the Dongying petroleum storage depot of SINOPEC. The OS was filtered and dried at 105 °C overnight as the precursor of this study. The OS precursor contains 32.2% of oil organic components, 16.6% of moisture, and the rest is solid residue. All the other chemical reagents used in this work were of AR grade and used as received without further purification. 

### 2.2. Preparation of MPCs

**Figure 1:**
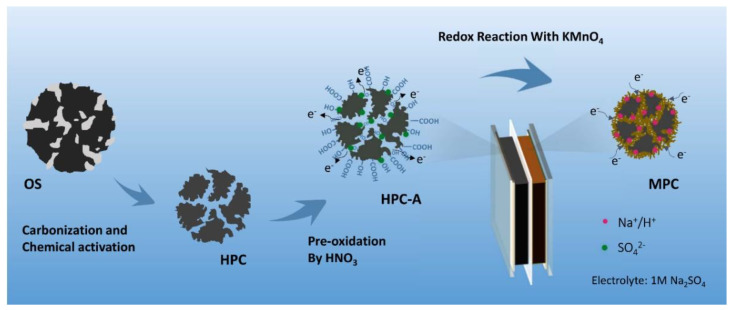
Schematic procedure for the fabrication of MPC electrode materials.

The preparation process of MPC is illustrated in Figure 1. First, OS-derived HPC material was prepared by carbonization and the chemical activation method according to our previous work [39]. Briefly, oily sludge was carbonized at 700 °C under N_2_ flow. After being washed by HF to remove inorganic impurities, the carbon residue was activated by KOH at a mass ratio of 1:3 under 700 °C for 2 h. Second, the as-received HPC was pre-oxidized in 2 M HNO_3_ for 6 h to obtain HPC-A. Then, 200 mg HPC-A and 790 mg KMnO_4_ powder were ultrasonically mixed in 100 mL of pure water in a flask. The flask was heated and refluxed at different times at 70 °C in a water bath under intense stirring. The precipitates were filtered out and rinsed with pure water for several times until the filtrate became colorless. The MPC samples synthesized under different refluxing times of 1 h, 3 h, and 5 h were named MPC-A-1, MPC-A-3, and MPC-A-5, respectively. As for a control sample, MPC-3 sample was synthesized following the same procedure, except that HPC was used instead of HPC-A.

### 2.3. Characterization

The morphology of as-prepared materials was analyzed by scanning electron microscopy (SEM, JSM-6700F, JEOL, Tokyo, Japan). Transmission electron microscopy (TEM) and energy dispersive spectrum (EDS) mapping images of samples were acquired on a JEM-2100UHR (JEOL, Tokyo, Japan) at an accelerating voltage of 100 kV. X-ray diffraction (XRD) patterns were recorded by PANalytical X’Pert Pro (Panalytical, Almelo, Netherland) with Cu Kα radiation (λ = 1.54178 Å). The surface chemical states of samples were characterized by X-ray photoelectron spectra (XPS) on an ESCALAB MK II (Thermo Scientific, Waltham, MA, USA) with Mg Kα (hυ = 1253.6 eV) as the excitation source. The pore structures were tested by a Multipoint N_2_ adsorption–desorption experiment on an automatic Micromeritics ASAP 2020 (Micromeritics, Norcross, GA, USA) analyzer at 77 K. The specific surface area was calculated by the BET method and the pore size distribution was generated from the desorption branch of the isotherm by the non-local density functional theory (NLDFT) method.

### 2.4. Electrochemical Measurements

To fabricate the working electrode, as-prepared MPCs samples were first ground below 45 μm and then mixed with carbon black and polyvinylidene-fluoride (PVDF) with a ratio of 8:1:1 (active material:conductive agent:binder) in N-methyl-2-pyrrolidone (NMP) to form a homogeneous slurry. Then, the resulting slurry was coated on nickel foam as a 1 cm × 1 cm sheet, followed by drying at 80 °C overnight. The mass loading of active materials on electrodes was controlled at about 2.8, 3.0, 2.8, 2.6, and 4.2 mg cm^−2^ for the MPC-A-1, MPC-A-3, MPC-A-5, MPC-3, and HPC-A electrodes, respectively.

Cyclic voltammetry (CV) and galvanostatic charge/discharge (GCD) electrochemical tests were carried out on a CHI660E electrochemical workstation. Electrochemical impedance spectroscopy (EIS) were performed on the Gramy Reference 600 (Shanghai Chenhua Science Technology Corp., Ltd., Shanghai, China) with a frequency range of 10^5^ to 10^−2^ Hz at an open circuit voltage. For typical three-electrode system measurements, an Ag/AgCl electrode and platinum sheet were employed as the reference and counter electrodes, respectively. Potential range for the CV and GCD test was from 0 to 1 V. For two-electrode system measurements, the MPC and HPC-A electrode was used as the cathode and anode, respectively, and then separated by a thin polypropylene film to fabricate the ASC device. The electrolyte was a 1 M Na_2_SO_4_ solution for both three-electrode and two-electrode systems.

The charge balance and the mass ratio of the cathode and anode in the ASC device and the detailed calculation of the specific capacitance of the electrodes and the energy density and power density of the as-assembled ASC device are shown in the Supplementary Materials.

## 3. Results and Discussion

### 3.1. Characterizations of MPC Samples

**Figure 2:**
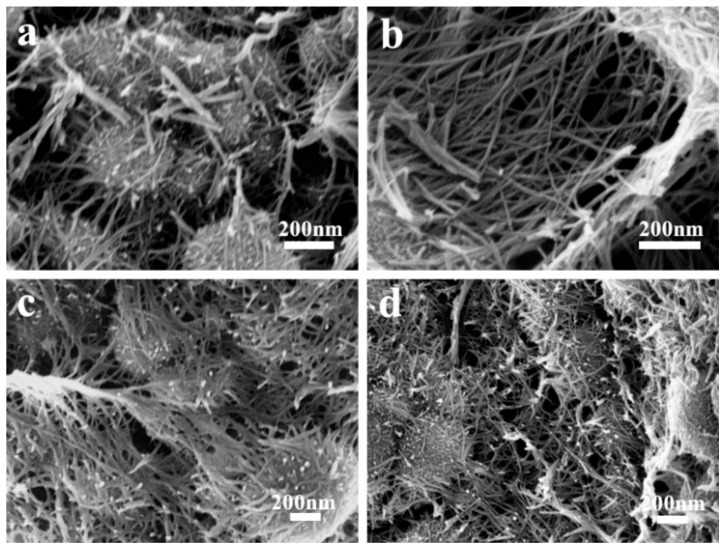
SEM images of the MPC-A-1 (**a**), MPC-A-3 (**b**), MPC-A-5 (**c**), and MPC-3 (**d**).

As shown in Figure S1a,b, a cross-linked porous structure could be found in the carbon skeleton in HPC-A, which maintains its hierarchical porous structure of HPC (Figure S1c,d) [39] after oxidation treatment by HNO_3_. The SEM images of MPCs are given in Figure 2 to analyze the structure details of the materials. The porous structure in the HPC-A substrate that provides space for MnO_4_^−^ ions to enter the interior and be reduced to MnO_2_ on the interior and exterior surfaces follows the equation [40]:

$${4\;\mathrm{MnO}}_{4}^{-}{+3\;C+H}_{2}O\;\to {\;4\;\mathrm{MnO}}_{2}{+\mathrm{CO}}_{3}^{2-}{+2\;\mathrm{HCO}}_{3}^{-}$$

In addition, the content and morphology of MnO_2_ in the as-received MPC could be controlled by varying the reaction time. As shown in Figure 2a, a unique “nest-like” porous structure with compact MnO_2_ nanowires with anisotropic growth could be observed on the surface of the carbon matrix in MPC-A-1. With an increase in reaction time, the diameters of MnO_2_ nanowires were increased, as shown in the SEM images of MPC-A-3 (Figure 2b), which made the “nest” more tight. As for MPC-A-5 (Figure 2c), the hierarchical porous structure of the HPC-A substrate was almost completely covered by the surface MnO_2_ nanostructure due to the over-reaction between carbon and KMnO_4_. Moreover, some egg-like structure could be observed in all of these samples, which may have been caused by the shrinking of the defective carbon materials when reacted with the strong oxidant KMnO_4_. Compared with MPC-A-3, the MPC-3 samples that were obtained under the same reaction time possessed shorter and more disordered arranged MnO_2_ nanowires that nearly could not form a “nest” (Figure 2d). This shows that the HPC-A substrate could make MnO_2_ nanowire anchoring more uniform, and effectively promote the growth of MnO_2_ nanowires during the following redox reaction. The MPC-A-3 possesses the structure of stable MnO_2_ nanowires wrapped on a HPC substrate, which can promote the pseudo capacitive of MnO_2_ as well as EDLC effect of porous carbon to increase the capacitance performance.

**Figure 3:**
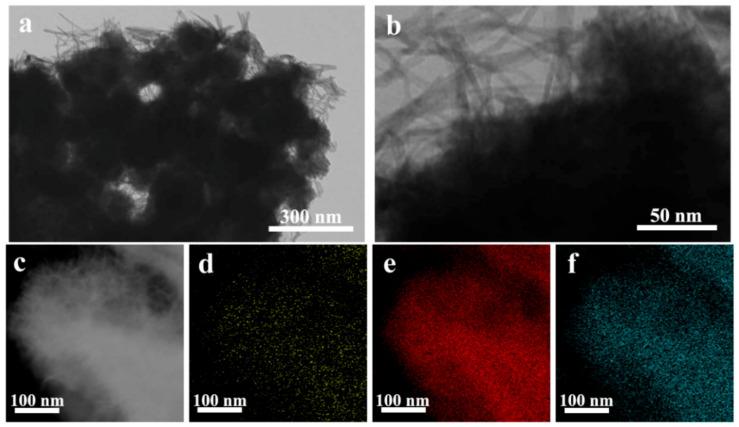
TEM images of the MPC-A-3 composite material (**a**–**c**) with corresponding elemental mapping images of C (**d**), Mn (**e**), and O (**f**).

TEM images of MPC-A-3 also showed a nest-like structure, where nanowires with a diameter below 10 nm were covered on the surface of the HPC-A substrate in Figure 3a,b. Furthermore, the results of the corresponding X-ray elemental mapping images of C, Mn, and O (Figure 3c–f) demonstrate the co-existence of these elements. Combined with the above SEM images, the MnO_2_ nanowires were successfully embedded in the HPC structures by the above method.

**Figure 4:**
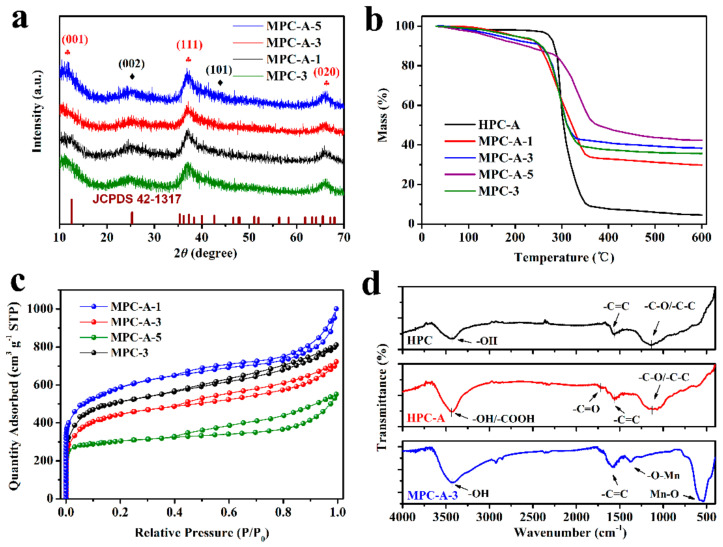
XRD patterns (**a**), TGA analyses (**b**), and N_2_ adsorption–desorption isotherms (**c**) of the MPC materials, and the FTIR spectra (**d**) of HPC, HPC-A, and MPC-A-3.

The composition of the samples was examined through the XRD test. As shown in Figure S1e, the HPC-A sample had a disordered and defective graphitic structure as evidently illustrated by the appearance of two broad peaks of (002) and (101) diffraction planes. The XRD patterns of MPCs are shown in Figure 4a, in which the broad diffraction peaks around 26° and 44° indicate the presence of amorphous graphitic carbon in the composites [41], while the characteristic peaks at 12°, 37°, and 66° correspond to the (001), (111), and (020) crystal planes of birnessite-type MnO_2_ (JCPDS 42-1317) [42], respectively. It was noticed that the intensity of the diffraction peak at 37° gradually increased with the reaction time due to the greater amount of MnO_2_ in the composites.

TGA tests were performed in an air atmosphere from 25 to 600 °C to calculate the loading amount of MnO_2_ in different MPCs. As shown in Figure 4b, the total weight loss of the HPC-A material was 95.45% and the remaining residue was mainly the unburned inorganic impurities. In addition, the weight loss of MPC-A-1, MPC-A-3, and MPC-A-5 was 70.24%, 61.72%, and 57.61%, respectively, which decreased with the increase in the reaction time. It is clear that the weight losses of the MPCs are mainly due to the combustion of carbon components. The MnO_2_ loading amount can be calculated by the difference of weight loss between HPC-A and MPCs. Therefore, the mass percentage of MnO_2_ contained in the MPC-A-1, MPC-A-3, and MPC-A-5 composites can be calculated as 26.41%, 35.34%, and 39.64%, respectively, which indicates that a longer reaction time was beneficial to MnO_2_ loading.

Combined with the observations in the SEM and TEM images, different MnO_2_ loading amounts could affect the porosity of MPCs. The N_2_ adsorption–desorption results of MPCs and HPCs are illustrated in Figure 4c and Figure S1f, respectively. All the samples exhibited a typical combined I/IV type isothermal with an obvious H4 hysteresis loop [43]. As is well-known, the sharp increases in adsorption capacity at low relative pressure are related to the micropores, while the relatively slower increases are caused by the capillary condensation of N_2_ in the mesopores of the sample. This means that the surface MnO_2_ loading did not change the basic skeleton structure of the total materials.

The pore size distributions of the MPCs are shown in Figure S2, and the pore structure parameters are listed in Table S1. The specific surface area (SSA) of the MPC sample is closely related to the reaction time. With the increase in reaction time from 1 h to 3 h and 5 h, the SSA decreased from 1957.4 to 1437.1 and 938.4 m^2^ g^−1^, respectively, which mainly resulted from the pore blocking. The total pore volume decreased from 1.397 to 0.775 cm^3^ g^−1^, which means that the MnO_2_ nanostructure is generated and filled in the pores of HPC-A. The increase in loading amount would cause more blockage of pores.

To further investigate the effect of pre-oxidation on MnO_2_ loading, FTIR analyses were performed, as shown in Figure 4d. The peaks of around 1100 cm^−1^, 1580 cm^−1^, and 3440 cm^−1^, which represent –C–C/–C–O, –C=C, and –OH, can clearly be seen in the spectra of the origin HPC sample, respectively. After being oxidized by HNO_3_, it was found that the characteristic peak derived from symmetric carboxyl appeared at 1680 cm^−1^, which indicates the acidic oxygen-containing surface functional groups’ oxidation treatment [44]. The FTIR spectra for MPC-A-3 shows the vibration of the Mn–O bond at the wavelength of 547 cm^−1^ in the fingerprint region. Moreover, it is known that the positions of symmetric –COO stretching bonds considerably vary in the range of 1300–1450 cm^−1^ in metal complexes depending on the coordination structures [45]. The appearance of a new peak at 1380 cm^−1^ is indicative of the symmetric carboxylate stretching mode, which also represents the existence of MnO_2_. Furthermore, the peak at 1680 cm^−1^ assigned to –C=O in HPC-A showed a red shift in MPC-A-3. This indicates that the double bond between carbon and oxygen on the HPC-A substrate becomes longer and weaker due to the formation of the electrostatic ionic bond with MnO_2_.

**Figure 5:**
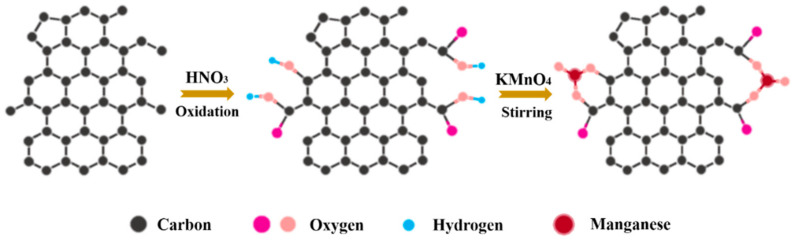
Scheme of the possible preparation mechanism of MPC materials.

The preparation mechanism of MPC materials was analyzed according to the above characterization results. As shown in Figure 5, abundant carboxyl and hydroxyl groups were produced on the surface of HPC-A. On one hand, these oxygen-containing functional groups made the reaction solution acidic, which could enhance the oxidation effect of KMnO_4_ and increase the reaction depth of KMnO_4_ and carbon [46]. On the other hand, these groups were beneficial in the generation of stronger chemical bond interactions between newly formed MnO_2_ and the surface of the carbon substrates. The nanowires were formed via head-to-head overlapping of nucleated MnO_2_ nanoparticles [47]. The head-to-head overlapping is possible because the bonding sites on the HPC surface are quite small, and the high number of coordination sites trigger the preferential condensation of the nucleated MnO_2_ nanoparticles into long ultrathin nanowires. Then, the slow growth and reduction in the MnO_2_ nanowires proceeded simultaneously during the reaction. Finally, nanowires led to further growth, aggregation, and assembled into a 3D nest-like structure due to the narrow space in the porous carbon substrates. Last but not least, the large SSA of the HPC-A substrate could provide a large number of sites for the embedding of MnO_2_ nanowires.

### 3.2. Capacitance Performance of MPCs

**Figure 6:**
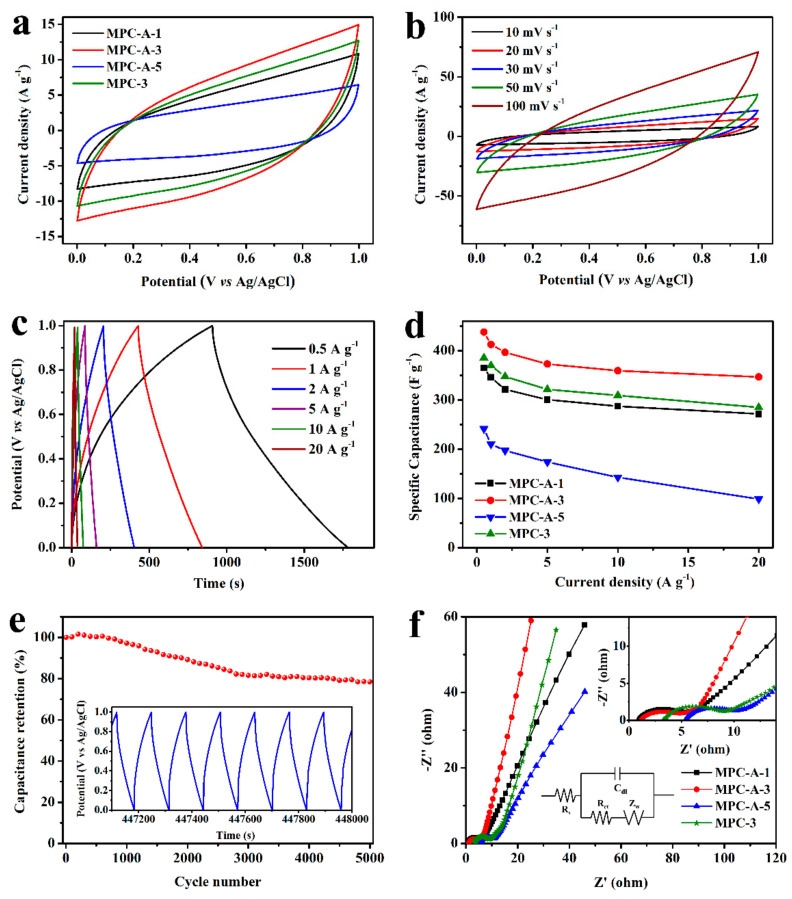
CV curves of different MPC material sat a scan rate of 20 mV s^−1^ (**a**). CV curves of MPC-A-3 at different scan rates (**b**). GCD curves of MPC-A-3 at different charge/discharge current densities (**c**). Rate capability of different MPCs material (**d**). The charge/discharge cycling ability (**e**) of MPC-A-3, the inset is the GCD curves after 3000 cycles. Nyquist plots of the different MPCs material (**f**), the inset is the enlarged image of the high frequency zone.

The electrochemical performance of all MPCs samples was first investigated in a three-electrode system in a Na_2_SO_4_ aqueous solution at room temperature. The representative CV curves recorded at a scan rate of 20 mV s^−1^ and a potential window from 0 to 1 V are shown in Figure 6a. All curves displayed an approximate rectangular shape, demonstrating the typical EDLC effect of electrode materials. Although redox peaks could not be clearly found in the CV curves, this electrochemical signature is consistent with the pseudo-capacitance nature of MnO_2_, which arises from reactions that are faradaic in origin, involving the passage of charge across the double layer [13]. This pseudo-capacitance is based on the insertion of cations (H^+^ or Na^+^) from the electrolyte with MnO_2_ upon reduction followed by de-insertion upon oxidation:

MnO_2_ + H^+^ + e^−^ → MnOOH

MnO_2_ + Na^+^ + e^−^ → MnOONa

Among these curves, the MPC-A-3 electrode revealed the largest area of the CV triangle, suggesting its highest capacitance value, which was due to the large surface area, optimal porous structure, and MnO_2_ loading. Compared with the MPC-3 sample, a higher capacitance value of MPC-A-3 means that the pre-oxidation treatment of the carbon substrate is beneficial to the capacitance performance of the composites. This is because the HPC-A substrate provides more sites for MnO_2_ loading and regulates the growth of MnO_2_ nanowires in the axial direction. The uniform distribution of MnO_2_ on the HPC skeleton promotes the transfer of electrolyte ions, which improves the capacitance performance. In addition, the capacitance of MPC-A-x first increased with reaction time and then decreased because the overloaded MnO_2_ would lead to the blockage and destruction of the pore structure of composites, which inhibits the acceptance of capacitance active sites. The CV curves of MPC-A-3 at different scan rates in Figure 6b still exhibited a symmetrical shape even at a high scan rate of 100 mV s^−1^, demonstrating a faster and reversible charging and discharging process. Similar trends can be found in the CV curves of MPC-A-1, MPC-A-5, and MPC-3 at different scan rates (Figure S3). The outstanding electrochemical capacitance performance of MPCs can be attributed to the hierarchical porous structure with large SSA and unblocked ion transport path. The reproducibility performance of the results were tested by a typical CV test. As shown in Figure S4, the shape of the CV curves showed no significant changes compared with the original tests with the differences under 5%.

The C_s_ of MPCs was investigated by GCD tests. The GCD curves of MPC-A-3 presented in Figure 6c and that of the other MPCs (Figure S5) exhibited similar triangular and symmetrical forms. The C_s_ of the MPC electrodes was calculated from these GCD curves and is shown in Figure 6d. The C_s_ of MPCs followed the order of MPC-A-3 > MPC-3 > MPC-A-1 > MPC-A-5 at each current density. The C_s_ of MPC-A-3 could reach 437.9 F g^−1^ (1.31 F cm^−2^) at the current density of 0.5 A g^−1^ (1.5 mA cm^−2^). In general, with the increase in current densities, the capacitance will decrease along with a lower amount of electrolyte ions occupying the active sites. The C_s_ of MPC-A-3 could maintain 346.6 F g^−1^ (1.04 F cm^−2^) at 20 A g^−1^ (60 mA cm^−2^) with the rate capability of 79.2%, which is much higher than MPC-A-1 (74.4%), MPC-A-5 (40.5%), and MPC-3 (73.8%). It is worth noting that MPC-3 showed a slightly worse rate capability than MPC-A-3 despite the same synthesis time. This could be due to a small number of MnO_2_ nanowires possibly falling off from the surface of the MPC-3 at the high charging/discharging current densities, which are caused by the weaker binding effort between MnO_2_ nanowires and the HPC substrate without pre-oxidized treatment. Furthermore, for MPC-A-3, the cycling stability performance was evaluated by the GCD test at the current density of 5 A g^−1^ for 5000 cycles. As illustrated in Figure 6e, the capacitance increased slightly in the first 500 cycles due to the initial activation process of MnO_2_ materials for pseudo capacitance. After 5000 cycles, the capacitance retention remained at 78.5% of the initial value, indicating outstanding cycling stability. The inset in Figure 6e shows the GCD curves after the 3000th cycle. The symmetrical shape of the curves and the voltage drop were not changed significantly, although the total charging/discharging time became shorter, which related to worse capacitance performance. The good cycling stability of MPC-A-3 makes it prospective for the practical applications of a supercapacitor.

The Nyquist plots of the MPCs electrodes obtained by the EIS test are shown in Figure 6f, where the almost vertical curves at the low frequency are closely related to ionic diffusion resistance, and the semicircular at high-middle frequency is associated with charge transfer resistance [48]. Based on the equivalent circuit as inset in Figure 6f, the impedance data were fitted. It was observed that the semicircle increased in the order of MPC-3 (7.32 Ω) > MPC-A-5 (6.68 Ω) > MPC-A-1 (5.28 Ω) > MPC-A-3 (4.36 Ω), indicating the MPC-A-3 possesses the smallest interfacial charge-transfer resistance (R_ct_), which represents the optimal ionic diffusion capacity. The equivalent serials resistance (R_s_) of each electrode followed the order of MPC-A-5 (5.36 Ω) > MPC-3 (3.38 Ω) > MPC-A-3 (1.15 Ω) > MPC-A-1 (0.94 Ω). Here, R_s_ increased with the increase in the reaction time of preparation, which is probably because the increase in MnO_2_ loading can inhibit the conductivity of the capacitive active material. Additionally, MPC-A-3 showed a lower R_s_ than MPC-3, which is due to the fact that the MnO_2_ combination onto MPC-A-3 was more ordered. This verifies that the pre-oxidation process could effectively improve the growth of MnO_2_ nanowires on the HPC-A substrate.

**Figure 7:**
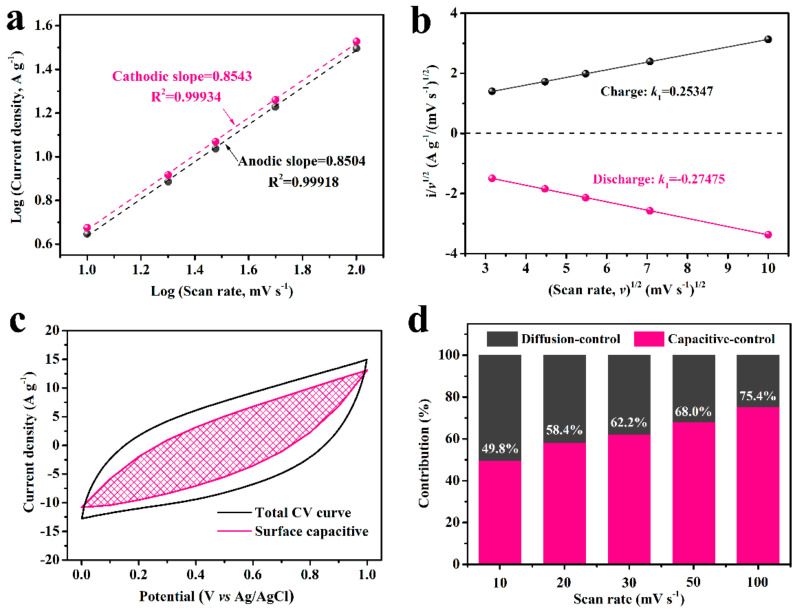
(**a**) Current vs. scan rate of the MPC-A-3 electrode at a potential of 0.5 V. (**b**) CV data at a potential of 0.5 V for the calculation of *k_1_*, (**c**) the storage contributions from the pseudo capacitance and EDLC at a scan rate of 20 mV s ^−1^. (**d**) Ratio of pseudo capacitance contributions in MPC-A-3 at different scan rates.

To elucidate the mechanism of the MPC composite electrode, the relative contributions of the EDLC and the pseudo capacitance to the electrochemical capacitance were further evaluated via CV curves. The current response at various potentials can be calculated by the following equation [49]:

$${i=a\;\times \;v}^{b}$$

where *i*(*v*) is the current density (mA); *ν* is the scan rate (mV s^−1^); and *a* and *b* are the coefficients. As it is known that the electrode shows a battery type with fully faradaic effect if *b* equals 0.5, then a total EDLC property has a *b* value of 1. The *b* values between 0.5 and 1 indicate the combination of the double layer capacitance and diffusion-controlled pseudo capacitance. As shown in Figure 7a, the cathodic and anodic *b* values for MPC-A-3 at a potential of 0.5 V are 0.8543 and 0.8504, respectively, implying the current response results from both surface EDLC capacitive and diffusion-controlled pseudo capacitive [50]. 

Moreover, the surface EDLC contribution and the pseudo capacitive contribution for MPC-A-3 electrode were also be calculated from the CV curves (Figure 6b) using the function [51]:

$$i=\;k_{1}v\;+k_{2}v^{1/2}$$

where *k*_1_ and *k*_2_ are the appropriate coefficient. The current response from the EDLC behavior could be determined by $k_{1}v$. *k*_1_ equals the slope of each curve at different potentials like that illustrated in Figure 7b. Taking $k_{1}v$ as the ordinate and corresponding potential as the abscissa, the EDLC capacitance is obtained. As shown in Figure 7c, for instance, the capacitive contributions from different processes at a scan rate of 20 mV s^−1^ were determined. The surface EDLC capacitive were marked as the mesh area, and the rest of the total capacitance was contributed by the diffusion-controlled pseudo capacitive. The relative contributions at different scan rates are summarized in Figure 7d. The contribution of the EDLC process increases from 49.8% to 75.4% for scan rates from 10 to 100 mV s^−1^. At low scan rates, the dominant current response arises from the diffusion-controlled process, which attributed to the pseudo capacitance of MnO_2_. At a higher scan rate, the faradaic process is inhibited and the surface EDLC process plays the major role in charge storage.

**Figure 8:**
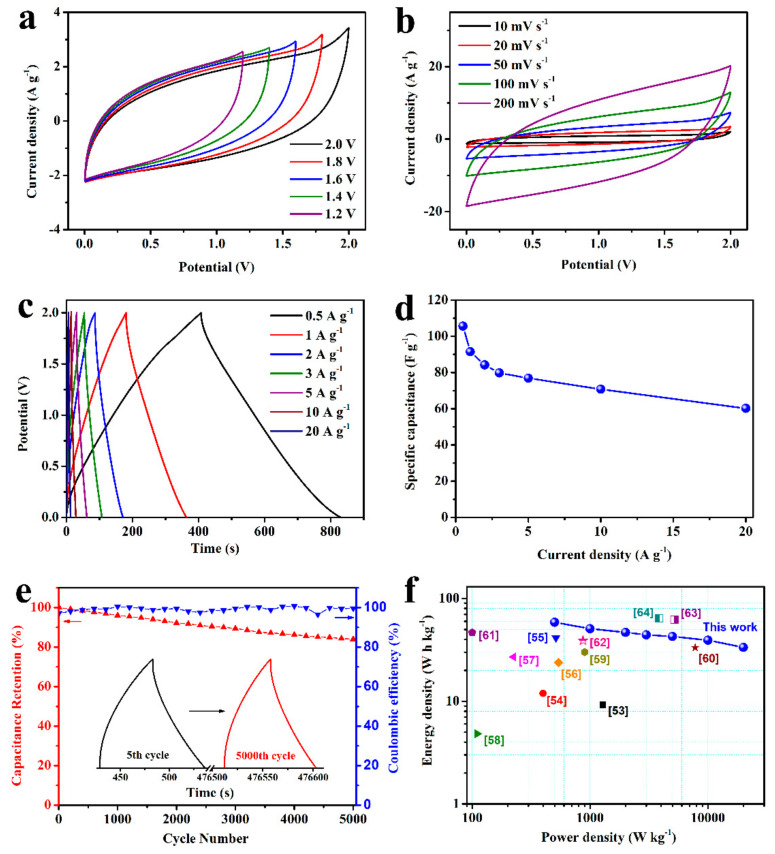
CV curves of the ASC devices in different potential windows (**a**) at 20 mV s^−1^ and CV curves at various scan rates in the working potential window of 2.0 V (**b**). GCD curves at different current densities from 0.5 to 20 A g^−1^ (**c**) and C_s_ calculated from the GCD curves (**d**). Cycling durability and coulombic efficiency of ASC at a current density of 3 A g^−1^ (**e**). Ragone plot of the as-assembled ASC devices (**f**).

To further evaluate the practical application of the MPC materials, ASC devices with extended working voltages was assembled with MPC-A-3 as the cathode and HPC-A as the anode. The electrochemical result of HPC-A is shown in Figure S6, which illustrates typical capacitance properties. According to the CV comparison in Figure S7, The optimal mass ratio of the cathode and anode was calculated as 0.68 [52]. As shown in the CV curves of this ASC device in Figure 8a, the potential window could be enlarged to 0–2.0 V. Nearly symmetric shape CV curves were obtained at different scan rates in the voltages of 2.0 V (Figure 8b). Afterward, GCD curves at different current densities with the voltage of 2.0 V are shown in Figure 8c. The curves exhibited a nearly linear potential-time behavior without significant voltage drop, suggesting rapid charging/discharging performance, which is consistent with the results of the CV tests. The specific capacitance can be calculated as 105.6, 91.5, 84.2, 79.8, 76.9, 70.8, and 60.2 F g^−1^ at 0.5, 1, 2, 3, 5, 10, and 20 A g^−1^ based on the total mass of two electrodes, indicating a good rate capability (Figure 8d). The area specific capacitance was 0.76, 0.66, 0.61, 0.57, 0.55, 0.51, and 0.43 F cm^−2^ at 3.6, 7.2, 14.4, 21.6, 36, 72, and 144 F cm^−2^, respectively. Moreover, the ASC device could undergo 5000 cycles with a capacitance retention of 83.9% and excellent coulombic efficiency (Figure 8e). The insets of Figure 8e illustrate the 5th and 5000th cycle GCD curves during the cycle stability test. The shape of the curves barely changed, although the total charging/discharging time was decayed due to the structure damage of the electrode after a long-term duration. Meanwhile, the energy densities and power densities of ASC were calculated and compared with the other reported data on the Ragone plot in Figure 8f. The maximum energy density of 58.67 W h kg^−1^ was obtained at the corresponding power density of 498.8 W kg^−1^, which still retained 33.44 W h kg^−1^ at 20 kW kg^−1^. These superior energy density properties are competitive with the reported MnO_2_-based supercapacitor devices listed in Table 1.

## 4. Conclusions

In summary, an oily sludge-based MnO_2_ nanowires/HPC composite electrode was successfully fabricated through an easy method. The MPC-A-3 electrode material delivered a high specific capacitance of 437.9 F g^−1^ at the current density of 0.5 A g^−1^, favorable rate capability of 79.2% retention at 20 A g^−1^, and long-term cycle stability. These good electrochemical properties could be attributed to the unique nanostructures of the MPC composite, where the HPC substrate improved conductivity and high surface area for EDLC behavior, and MnO_2_ nanowires provided enhanced pseudo-capacitance. Moreover, an ASC device assembled with MPC-A-3 as a cathode while the HPC as anode could be cycled reversibly at a prolonged voltage of 0–2.0 V. This ASC device maintained 83.9% of initial capacitance after 5000 cycles with an energy density of 58.67 W h kg^−1^ at a corresponding power density of 498.8 W kg^−1^. These results demonstrate that our MnO_2_ nanowire/HPC composite material from OS would be a promising electrode in practical supercapacitor applications.

## Figures and Tables

**Table 1:** Comparison of the energy density with other reported ASC.

Related ASC Devices	Current Collector	Potential Window (V)	Electrolyte	Specific Capacitance (F g^−1^)	Energy Density (W h kg^−1^)	Reference
MPC//HPC	Ni foam	(0–2)	1 M Na_2_SO_4_	105.6 at 0.5 A g^−1^	58.67 at 498.8 W kg^−1^	This work
MnO_2_@R//porous carbon	Ni foam	(0–2)	1 M Na_2_SO_4_	25.8 at 0.5 A g^−1^	9.2 at 1283.7 W kg^−1^	[53]
MnO_2_/CNTs//AC	Ni sheet	(0–1.6)	2 M KOH	-	11.95 at 398.3 W kg^−1^	[54]
MnO_2_/rGO/CNTs//AC	Carbon foam	(0–1.8)	1 M Na_2_SO_4_	54.4 at 0.5 A g^−1^	41.6 at 513.7 W kg^−1^	[55]
MnO_2_@TiC/C//AC	-	(0–1.5)	1 M Na_2_SO_4_	76.5 at 0.5 A g^−1^	23.9 at 540 W kg^−1^	[56]
MnO_2_/CNTs//AC	Al foil	(0–1.8)	1 M Na_2_SO_4_	60 at 0.25 A g^−1^	27 at 225 W kg^−1^	[57]
MnO_2_/CNT//PPy/CNT	-	(0–1.8)	1 M Na_2_SO_4_	-	4.82 at 110 W kg^−1^	[58]
MnO_2_/HNPC//HNPC	Ni foam	(0–1.8)	2 M Ca(NO_3_)_2_	66.9 at 1 A g^−1^	30.1 at 900 W kg^−1^	[59]
MnO_2_/HC//HC	Ti foil	(0–2)	1 M Na_2_SO_4_	-	33.3 at 7800 W kg^−1^	[60]
MnO_2_/GO//HPC	Ni foam	(0–2)	1 M Na_2_SO_4_	84 at 0.1 A g^−1^	46.7 at 100 W kg^−1^	[61]
α-MnO_2_//α-MnO_2_	Ni foam	(0–2)	1 M KOH	139.9 at 0.5 A g^−1^	38.9 at 870.3 W kg^−1^	[62]
MnO_2_/CN/PVDF// MnO_2_/CN/PVDF	Carbon fiber paper	(0–2)	0.5 M Na_2_SO_4_	-	64.39 at 3870 W kg^−1^	[63]
MnO_2_//Carbon	Ni foam	(0–2)	0.5 M K_2_SO_4_	115 at 1 mA cm^−2^	62.3 at 5200 W kg^−1^	[64]

## Data Availability

The data presented in this study are available on request from the corresponding author.

## Supplementary Materials

The following are available online at https://www.mdpi.com/article/10.3390/nano11102715/s1, Figure S1: The SEM (a) and TEM images (b) of HPC; SEM (c) and TEM (d) images of HPC-A; the XRD patterns of HPC and HPC-A (e), and the N_2_ adsorption–desorption isotherm (f) of the HPC and HPC-A samples; Figure S2: Pore size distribution and cumulative pore volume curves of MPC-A-1 (a), MPC-A-3 (b), MPC-A-5 (c), and MPC-3 (d) calculated by the NLDFT method; Figure S3: CV curves of MPC-A-1 (a), MPC-A-5 (b), and MPC-3 (c) at different scan rates; Figure S4: Reproduced CV tests (a) of the MPC-A-3r at different scan rates, the comparison of the CV curves (b) of the reproduced tests and the original typical tests; Figure S5: GCD curves of MPC-A-1 (a), MPC-A-5 (b), and MPC-3 (c) at different charge/discharge current densities and the comparison of GCD curves (d) of different MPCs samples at a current density of 2 A g^−1^; Figure S6: The CV curves of the HPC-A anode at different scan rates (a) and the GCD curves of the HPC-A anode at different current densities (b); Figure S7: Comparative CV curves of MPC-A-3 and HPC-A electrodes; Table S1: Pore structure parameters of MPCs.
