# Supplementary material for: Nest-Like MnO2 Nanowire/Hierarchical Porous Carbon Composite for High-Performance Supercapacitor from Oily Sludge
Source: Nanomaterials (Basel). 2021 Oct 14;11(10):2715. doi: 10.3390/nano11102715 (PMC8537434; doi:10.3390/nano11102715)
Supplement: Supplementary file 1 [file nanomaterials-11-02715-s001.zip › nanomaterials-1388584-supplementary.pdf]

Supplementary Materials

Nest-Like MnO_2 Nanowire/Hierarchical Porous Carbon
Composite for High-Performance Supercapacitor from Oily Sludge

Xiaoyu Li [1,2,*], Dong Han [2], Zhiqiang Gong [3] and Zhenbo Wang [2]

[1] College of Mechanical and Electronic Engineering, Shandong University of Science and Technology, Qingdao 266590, China
[2] College of New Energy, China University of Petroleum (East China), Qingdao 266580, China; S16030382@s.upc.edu.cn (D.H.); wangzhb@upc.edu.cn (Z.W.)
[3] State Grid Shandong Electric Power Research Institute, Jinan 250003, China; gongzhiqiang@upc.edu.cn
* Correspondence: lixy2018@sdust.edu.cn

Calculation methods

The mass specific capacitance (C_s, F g^{-1}) of the single electrode could be calculated from the GCD curves based on the following equation:

$$C_s = \frac{I_s \Delta t}{\Delta V}$$

Where, I_s (A g^{-1}) is the discharge current density in active material mass loading, Δt (s) is the discharge time, ΔV (V) is the potential window.

The area specific capacitance (C_a, F cm^{-2}) of the electrode could be calculated as the following equation:

$$C_a = \frac{I_a \Delta t}{\Delta V}$$

Where, I_a (A cm^{-2}) is the discharge current density in effective area of the electrode, and the other symbols are the same as the above.

And for ASC device test, the specific capacitance could also be calculated as the above equations, by just taking the total mass of active materials on cathode and anode for C_s, and taking the overlapped effective area of cathode and anode for C_a, respectively.

The energy density (E, W h kg^{-1}) and power density (P, W kg^{-1}) of the ASC device could be calculated by the following equations,

$$E = \frac{C_s \Delta V^2}{2 \times 3.6}$$

$$P = 3600 \times \frac{E}{\Delta t}$$

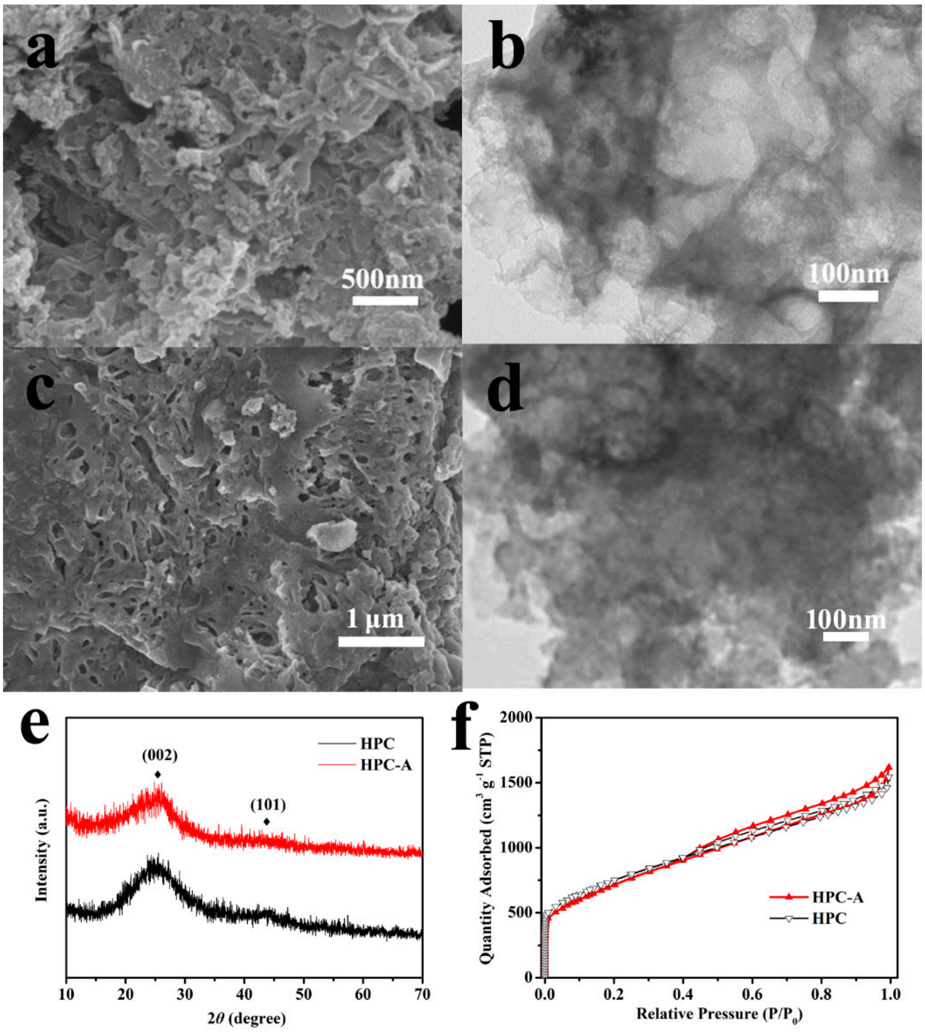

Figure S1. The SEM (**a**) and TEM images (**b**) of HPC, The SEM (**c**) and TEM (**d**) images of HPC-A, The XRD patterns of HPC and HPC-A (e), and the N_2 adsorption-desorption isotherm (f) of HPC and HPC-A samples

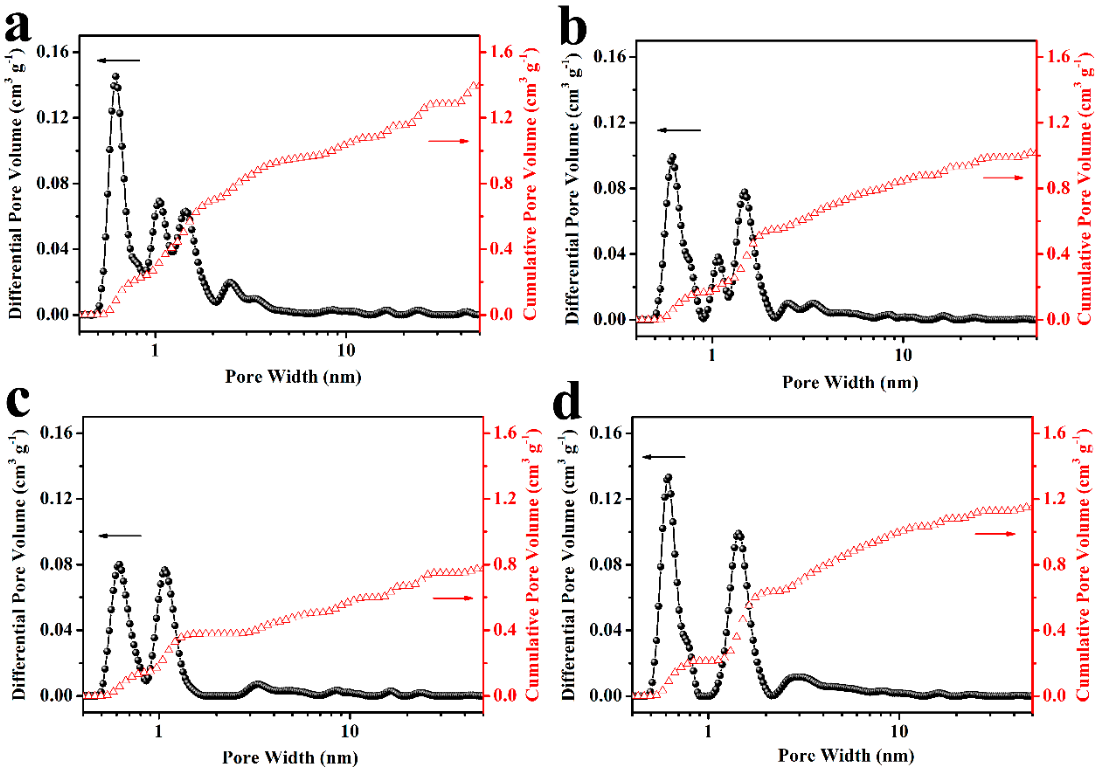

Figure S2. Pore size distribution and Cumulative pore volume curves of MPC-A-1 (**a**), MPC-A-3 (**b**), MPC-A-5 (**c**) and MPC-3 (**d**) calculated by NLDFT method

Table S1. Pore structure parameters of MPCs

Sample	S_{BET} [m^2 g^{-1}]	S_{mirco} [m^2 g^{-1}]	S_{meso} [m^2 g^{-1}]	V_{pore} [cm^3 g^{-1}]	V_{mirco} [cm^3 g^{-1}]	V_{meso} [cm^3 g^{-1}]
MPC-A-1	1957.4	1445.6	511.8	1.397	0.701	0.696
MPC-A-3	1437.1	1048.1	389	1.018	0.547	0.561
MPC-A-5	938.4	873.0	65.4	0.775	0.378	0.397
MPC-3	1638.8	1342	296.8	1.178	0.640	0.538

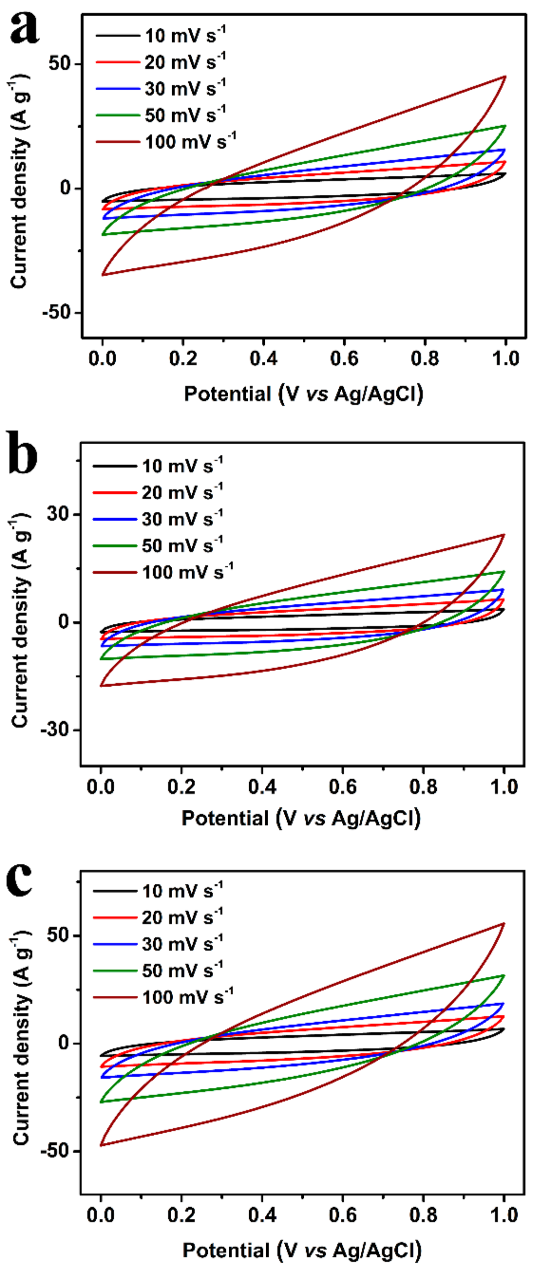

Figure S3. CV curves of MPC-A-1 (**a**), MPC-A-5 (**b**), MPC-3 (**c**) at different scan rates

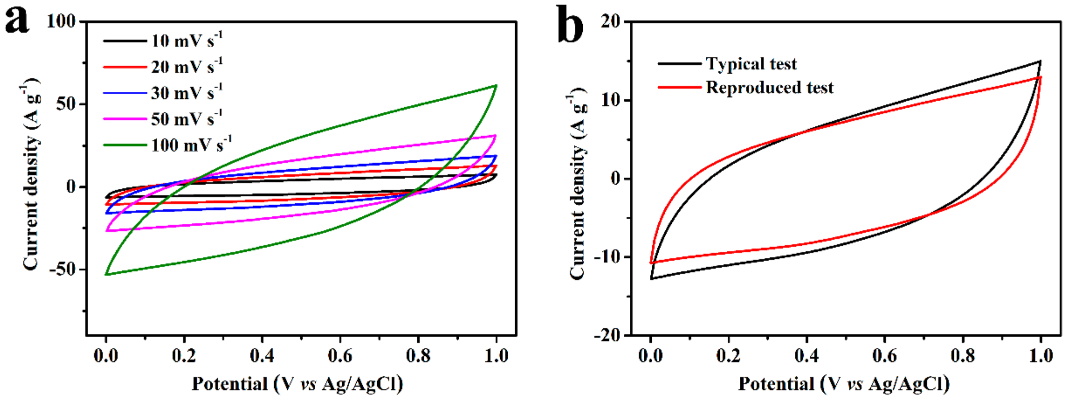

Figure S4. (a) Reproduced CV tests of the MPC-A-3r at different scan rates, (b) the comparison of the CV curves of the reproduced tests and the original typical tests.

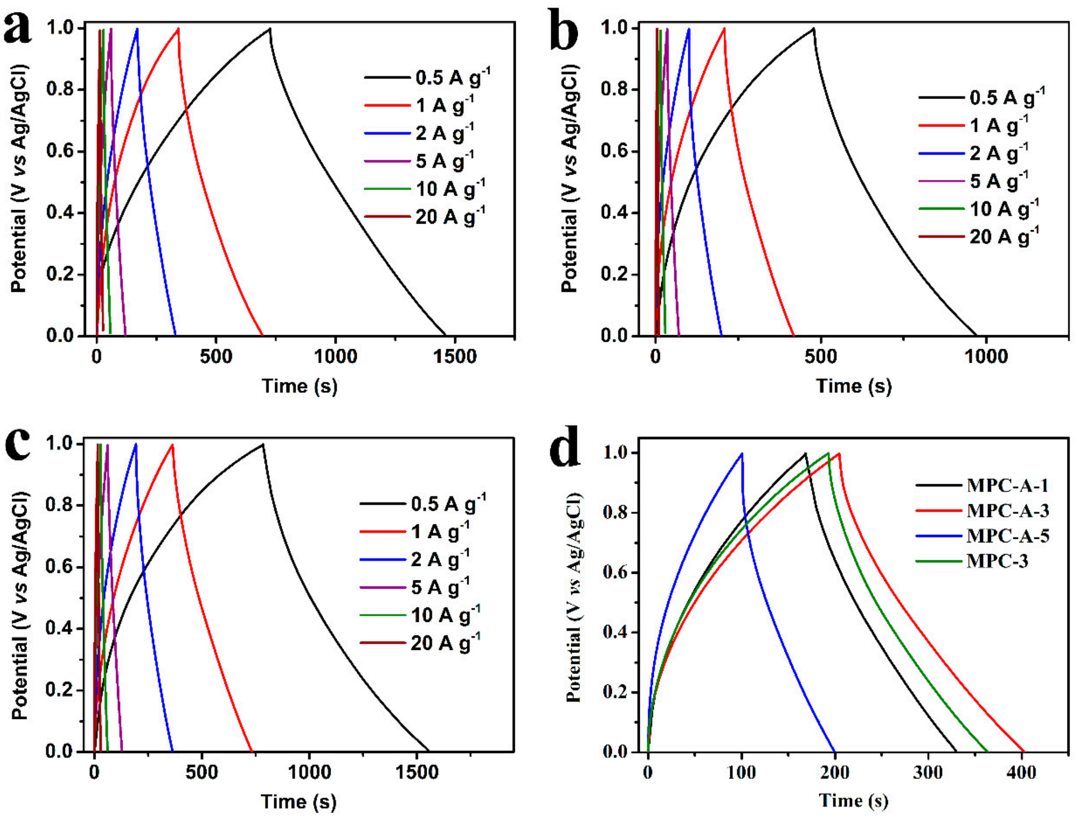

Figure S5. GCD curves of MPC-A-1 (**a**), MPC-A-5 (**b**), MPC-3 (**c**) at different charge/discharge current densities and the comparison of GCD curves (**d**) of different MPCs samples at a current density of 2 A g^{-1}

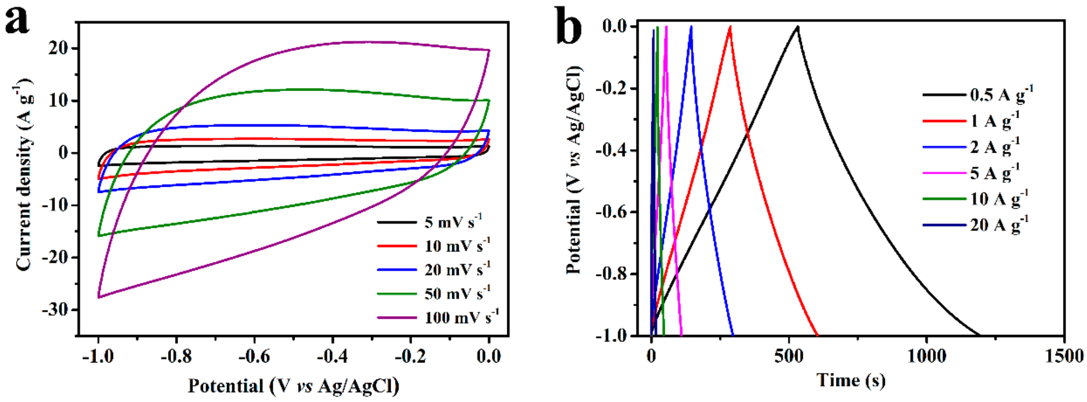

Figure S6. (**a**) The CV curves of HPC-A anode at different scan rates and (**b**) The GCD curves of HPC-A anode at different current densities.

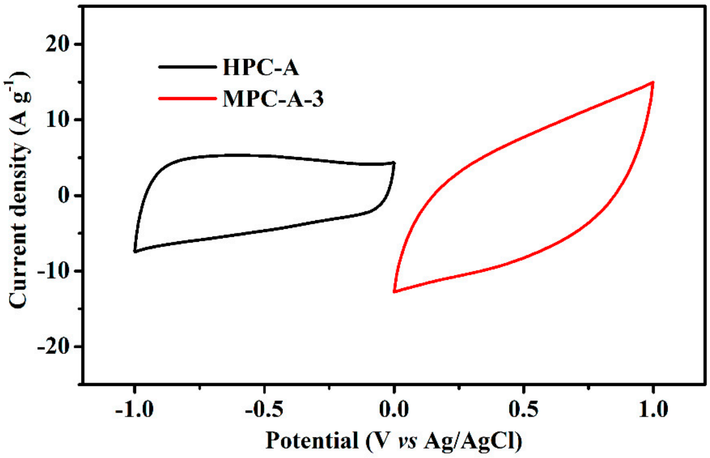

Figure S7. Comparative CV curves of MPC-A-3 and HPC-A electrodes

For an ASC device, charge storage on the cathode and anode will be balance and follow the relationship of $Q_{cathode}=Q_{anode}$. The mass ratio of active material on both electrodes for the optimum performance satisfied the following equation:

$$\frac{m_{cathode}}{m_{anode}}=\frac{C_{anode}\times V_{anode}}{C_{cathode}\times V_{cathode}}$$

The overlaid CV curves of MPC-A-3 and HPC-A measured on three electrode systems at a scan rate of 20 mV s^{-1} are illustrated in Figure S6. As observed from the curves, the potential window of HPC-A and MPC-A-3 is in the range of -1.0-0 V and 0-1 V, respectively. In addition, the specific capacitances of both HPC-A and MPC-A-3 could be calculated by the area of CV curves. Therefore, the calculated optimum mass ratio of the two electrodes was about 0.68 according to the above equation. The mass loading of MPC-A-1, MPC-A-3, MPC-A-5, MPC-3 and HPC-A as active materials on electrodes were about 2.8, 3.0, 2.8, 2.6 and 4.2 mg cm^{-2} in practice experiment, respectively.
